# Exploring the Emerging Association Between Immune Checkpoint Inhibitors and Thrombosis

**DOI:** 10.3390/jcm14103451

**Published:** 2025-05-15

**Authors:** Hassan Fawaz, Hasan Numan, Mohamad Hadi El Charif, Nicole Charbel, Sacha El Khoury, Joe Rizkallah, Amal El Masri, Arafat Tfayli, Firas Kreidieh

**Affiliations:** 1 Division of Hematology and Oncology, Department of Internal Medicine, American University of Beirut, Beirut P.O. Box 11-0236, Lebanon; hf74@aub.edu.lb (H.F.); hn72@aub.edu.lb (H.N.); me209@aub.edu.lb (M.H.E.C.); nc47@aub.edu.lb (N.C.); se147@aub.edu.lb (S.E.K.); axm09@mail.aub.edu (A.E.M.); at35@aub.edu.lb (A.T.); 2 Department of Diagnostic Radiology, American University of Beirut, Beirut P.O. Box 11-0236, Lebanon; jr56@aub.edu.lb

**Keywords:** immune checkpoint inhibitor, thrombosis, venous thromboembolism, arterial thrombosis, cancer-associated thrombosis, anticoagulation, immunotherapy

## Abstract

Immune checkpoint inhibitors (ICIs) have revolutionized cancer treatment, but their association with thrombosis presents significant clinical challenges. Patients with cancer already exhibit elevated risks for venous thromboembolism and arterial thrombosis, with treatment modalities like chemotherapy further exacerbating this risk. Emerging evidence suggests that ICIs contribute to thrombotic events through multifactorial mechanisms, including immune dysregulation, T cell activation, endothelial dysfunction, elevated tissue factor expression, and impaired fibrinolysis. Additional risk factors such as obesity, smoking, prior thrombotic events, and combination ICI therapy further increase thrombosis susceptibility. The literature reports varying incidence rates of ICI-associated thrombosis, with some studies indicating comparable risks to chemotherapy, while others highlight higher rates, particularly during the initial treatment phase. Management aligns with standard protocols for cancer-associated thrombosis, using low-molecular-weight heparin or direct oral anticoagulants, though optimal treatment duration and the role of prophylactic anticoagulation require further investigation. This review provides a comprehensive overview of the mechanisms, incidence rates, and clinical management strategies of ICI-associated thrombosis, emphasizing the importance of proactive risk assessment to optimize patient outcomes.

## 1. Introduction

Venous thromboembolism (VTE) and arterial thrombosis are significant complications of cancer and its treatment [1]. Along with infections, they are among the leading noncancer-related causes of death [2]. The risk of VTE is 12 times higher than in the general population, rising to 23 times in those receiving chemotherapy or targeted therapy [3]. Moreover, patients with cancer face an increased risk of arterial thrombosis, particularly in the short term after diagnosis. A large population-based study reported a six-month cumulative incidence of 4.7% for arterial thrombosis in patients with cancer, compared with 2.2% in matched controls [4].

Bleeding also carries an increased risk of mortality, particularly in patients with cancer receiving anticoagulation [5,6]. The 12-month cumulative incidence of clinically relevant bleeding in patients with cancer is 16.6%, with even higher rates in those with head and neck cancers [6]. This elevated risk is driven by multiple factors, including the malignancy itself, cancer-directed therapies, and comorbid conditions. Furthermore, the high risk of recurrent VTE in patients with cancer necessitates long-term anticoagulation, further increasing the likelihood of bleeding [7]. Therefore, reducing thrombosis risk to minimize the need for anticoagulation in this already high-risk population is essential.

Over the past decade, immune checkpoint inhibitors (ICIs) have transformed cancer treatment. These monoclonal antibodies target immune checkpoints, such as programmed cell death protein 1 (PD-1) and cytotoxic T lymphocyte-associated antigen 4 (CTLA-4), which normally suppress immune activity. Tumor cells often exploit these pathways to evade immune detection. By inhibiting these checkpoints, ICIs reactivate T cells, enabling them to recognize and eliminate tumor cells [8]. Although early phase III trials leading to their approval did not commonly report thrombosis as a side effect, real-world clinical experience has raised concerns, with studies reporting variable thrombosis rates [9]. Recent observational studies suggest that patients receiving ICIs may have a higher-than-expected risk of venous and arterial thromboses. Some studies have also associated thromboembolic complications during ICI treatment with poorer survival. However, whether this risk exceeds that associated with chemotherapy remains unclear [9].

In this review, we explore the emerging association between ICIs and thrombotic complications, focusing on potential mechanisms, reported incidence rates, and identified risk factors. We also discuss the clinical implications of these findings, current management strategies, and the need for further research to optimize patient outcomes while minimizing thrombotic risk.

## 2. Pathophysiology of Thrombosis

Thrombosis results from Virchow’s triad: venous stasis, endothelial injury, and hypercoagulability [10,11]. Reduced blood flow induces hypoxia, downregulating key antithrombotic proteins and creating a procoagulant environment that promotes thrombus formation. Venous thrombi consist of a platelet-rich white thrombus and a red blood cell- and fibrin-dense red thrombus [11]. Cancer markedly increases thrombosis risk, with VTE being a major cause of morbidity and mortality. Risk factors include tumor type and stage, patient history, and treatments such as surgery and chemotherapy, which may further elevate the risk of thrombosis [12].

Despite advances in ICIs over the past decade, their potential role in thrombogenesis remains poorly studied. Blocking immune checkpoint pathways such as PD-1/programmed death-ligand 1 (PD-L1) and CTLA-4 not only enhances T cell-mediated tumor suppression but also promotes inflammatory responses in vascular lesions, potentially leading to plaque rupture and thrombotic events such as acute coronary syndrome and cerebral infarction [13]. Therefore, a better understanding of the relationship between ICIs and thrombosis is needed.

## 3. Potential Mechanisms of ICI-Associated Thrombosis

**Figure 1:**
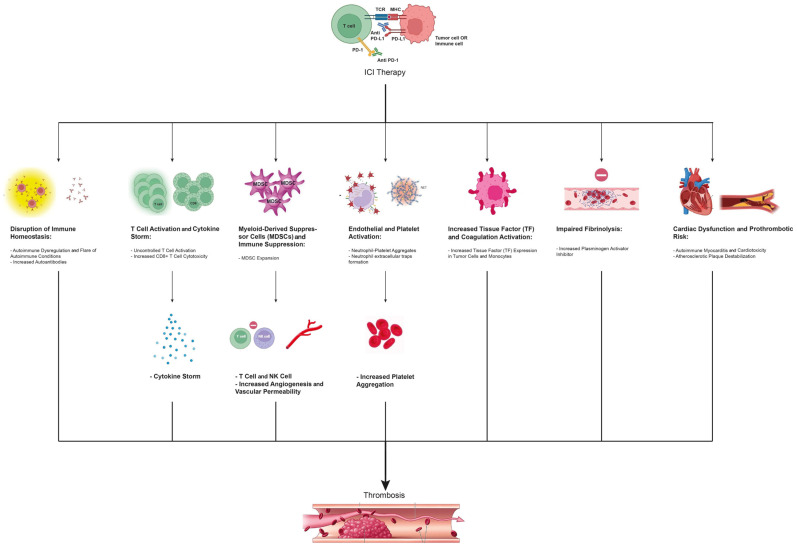
Mechanisms of immune checkpoint inhibitor-associated thrombosis. The figure illustrates key pathways through which ICIs contribute to thrombotic events. By targeting immune checkpoints such as PD-1/PD-L1, ICI therapy disrupts immune homeostasis, leading to autoimmune dysregulation and increased autoantibody production. Uncontrolled T cell activation triggers a cytokine storm and enhances CD8+ T cell cytotoxicity. MDSCs expand, promoting immunosuppression while increasing angiogenesis and vascular permeability. Endothelial and platelet activation, driven by neutrophil–platelet aggregates and NETs, further promotes platelet aggregation. ICIs also upregulate TF expression in tumor cells and monocytes, activating the coagulation cascade. Furthermore, impaired fibrinolysis secondary to elevated plasminogen activator inhibitor-1 levels contributes to clot persistence. Cardiac dysfunction, including autoimmune myocarditis and atherosclerotic plaque destabilization, further elevates thrombotic risk. These interconnected mechanisms collectively drive thrombosis in patients receiving ICI therapy. ICI, immune checkpoint inhibitor; MDSC, myeloid-derived suppressor cell; MHC, major histocompatibility complex; NET, neutrophil extracellular trap; NK, natural killer; PD-1, programmed cell death protein 1; PD-L1, programmed death-ligand 1; TCR, T cell receptor; TF, tissue factor.

Although the exact pathogenesis of ICI-associated thrombosis remains unclear, multiple mechanisms have been implicated, as illustrated in Figure 1. Cytokine activation triggers endothelial and platelet activity, and myeloid-derived suppressor cells (MDSCs) may also play a key role. In addition, T cell activation, increased tissue factor (TF) expression, PD-1 blockade, and autoimmune effects contribute to a prothrombotic state, underscoring the complexity of these mechanisms. These processes interact in a multifaceted manner, collectively increasing thrombotic risk. The following section analyzes the existing evidence on the mechanistic pathways underlying ICI-associated thrombosis, providing a comprehensive overview of its pathophysiology.

### 3.1. Disruption of Immune Homeostasis

ICIs are believed to disrupt immune homeostasis [14], inducing autoimmune responses and promoting thrombosis [15]. They have been associated with increased autoantibody levels, a key factor linked to the development of immune-related adverse events [16,17,18,19]. For example, ICI-treated patients can develop de novo autoimmunity such as autoimmune thyroiditis, hepatitis, or colitis, accompanied by high titers of autoantibodies. ICIs can also exacerbate preexisting autoimmune conditions, triggering flares as a result of immune activation, which disrupts immune tolerance and amplifies autoreactive immune responses. These flares, in turn, contribute to a proinflammatory state that promotes thrombosis [20,21,22,23].

The regulation of PD-L1 stability, influenced by proteins such as CKLF-like MARVEL transmembrane domain-containing proteins 4 and 6, is essential for maintaining immune homeostasis. Disruption of these molecules—as shown in experimental models—destabilizes PD-L1 expression and heightens immune system activity. This disruption has been linked to autoimmune diseases, including systemic lupus erythematosus and primary Sjögren’s syndrome, suggesting that ICIs may exacerbate autoimmune disorders [24].

Emerging evidence also suggests that ICIs may act as a trigger for antiphospholipid syndrome, an autoimmune thrombotic disorder characterized by the presence of antiphospholipid antibodies. Notably, there are case reports of patients developing catastrophic antiphospholipid syndrome—a severe, life-threatening form—while on ICI therapy. In one report, a patient receiving an anti-PD-1 ICI for gastric cancer developed catastrophic antiphospholipid syndrome with multiple thrombotic occlusions [25]. Similarly, another patient with metastatic melanoma treated with pembrolizumab experienced the onset of catastrophic antiphospholipid syndrome [26]. In both cases, the patients tested positive for antiphospholipid antibodies (such as anticardiolipin and anti-β2 glycoprotein I) and had no prior history of antiphospholipid syndrome, highlighting a potential direct link between ICIs and the development of this thrombotic autoimmune condition. These observations in patients show that immune homeostasis disruption by ICIs, leading to autoantibody production, can precipitate thrombosis in a clinical setting.

### 3.2. T Cell Activation

The PD-1/PD-L1 pathway functions as a “brake” that limits T cell activation and proliferation. Studies in PD-L1/L2-deficient mice have shown a greater atherosclerotic burden, increased numbers of activated CD4+ and CD8+ T cells within lesions, and elevated production of proinflammatory cytokines such as tumor necrosis factor [27,28]. Although PD-1 deficiency promotes the expansion of regulatory T cells, dominant proinflammatory T cell activation ultimately exacerbates atherosclerosis. This highlights the critical role of PD-1 signaling in maintaining peripheral tolerance by supporting regulatory T cell development and suppressing self-reactive T cells [29,30]. Moreover, PD-1 deficiency enhances the cytotoxic activity of CD8+ T cells, further amplifying inflammation and lesion formation in atherosclerosis-prone mice [28,29,30,31].

Although these findings come from animal models, they are informative and mirror what may occur in ICI-treated patients in terms of heightened T cell activity-producing systemic inflammation. In the context of thrombosis, activated T cells are a source of cytokines and can engage in crosstalk with the coagulation system. Therefore, the overactivation of T cells due to ICIs provides a link between cancer immunotherapy and thromboinflammatory processes [32].

### 3.3. Increased Tissue Factor Expression

One direct way that hyperactivated T cells can promote thrombosis is by inducing TF expression. TF is the primary initiator of the extrinsic coagulation pathway. ICI-activated T cells (particularly Th1-type CD4+ T cells) release interferon-gamma, a cytokine which can upregulate TF expression in various cells, including tumor cells and monocytes. Shim et al. demonstrated that T cell activation enhances thrombus formation in experimental models by increasing prothrombotic markers, including neutrophil extracellular traps (NETs) and circulating nucleosomes [33]. These findings align with research by Sato et al. [34], which showed that activated T cells during ICI therapy induce TF production in peripheral PD-L1-high monocytes, establishing a link between ICI therapy and coagulation activation. Together, these studies indicate that when ICIs drive strong T cell responses, a downstream effect is the upregulation of TF on cells, creating a potent trigger for the coagulation cascade.

This phenomenon parallels findings in other inflammatory conditions, such as inflammatory bowel disease, where T cells contribute to thrombotic events through TF-dependent thrombin production. In a murine model of inflammatory bowel disease, colitogenic CD4+ T cells expressing TF promote rapid plasma thrombin generation, resulting in a shortened clotting lag time and an increased peak thrombin level; interestingly, this TF-driven hypercoagulability can be attenuated by inhibiting disulfide isomerase—a cofactor of TF—with the flavonoid rutin [35]. This example illustrates how T cell-induced TF expression directly translates to a prothrombotic state. By analogy, the ICI-induced increase in TF expression is a major mechanism heightening thrombotic risk in patients with cancer receiving immunotherapy. TF, once expressed on cell surfaces, binds factor VII/VIIa and initiates the clotting cascade, leading to thrombin generation and fibrin clot formation [36]. Thus, while ICIs enhance antitumor immunity through T cell activation, they concurrently raise levels of TF in the circulation or tumor microenvironment, tipping the balance toward coagulation [33]. This underscores the complex balance between enhancing antitumor immunity and mitigating thrombotic complications in ICI therapy.

### 3.4. Myeloid-Derived Suppressor Cell Activity

MDSCs play a crucial role in modulating the immune response to cancer treatments, particularly ICIs. Known for their immunosuppressive properties, MDSCs contribute to ICI resistance and may also be involved in therapy-associated thrombosis. Elevated levels of MDSCs are often found in patients with cancer and can increase further during ICI therapy, especially in patients who do not respond well to treatment. These elevated levels have been linked to poor prognosis in patients with cancer receiving ICI therapy, as their presence not only reduces therapeutic efficacy but also increases susceptibility to VTE [37,38].

By suppressing T cells and natural killer cells, MDSCs create an immunosuppressive environment that reduces ICI efficacy, supports tumor growth and metastasis, and promotes thrombosis through several mechanisms. By interacting with and inhibiting T cells, MDSCs blunt adaptive immunity, but they also secrete various factors that influence the endothelium and blood coagulation. For instance, MDSCs release interleukin-8, a chemokine that not only attracts neutrophils but is associated with coagulation activation and VTE risk. MDSCs can increase vascular permeability and support aberrant angiogenesis in tumors [39,40,41], which can expose procoagulant surfaces and contribute to a thrombotic tendency, as leaky new blood vessels and endothelial disruption favor clotting. They can also interact directly with platelets and monocytes. Rolling et al. reported that MDSCs engage platelets via checkpoint molecule interactions, potentially leading to platelet activation or the release of TF-bearing microparticles from monocytes [42]. In essence, MDSCs promote immunosuppression and inflammation, further increasing thrombotic risk [43].

Clinically, higher MDSC levels have been linked to worse outcomes and higher thrombotic risk in patients receiving ICIs. In a study by Roopkumar et al., patients who developed VTE during ICI therapy had significantly higher pretreatment levels of MDSCs, interleukin-8, and soluble vascular cell adhesion molecule-1, suggesting their potential as predictive biomarkers for thrombosis in these patients. The study also identified an interleukin-8- and MDSC-driven pathway involved in VTE pathogenesis, highlighting the inflammatory role of MDSCs in establishing a prothrombotic environment [44].

In summary, MDSCs—through their immunosuppressive yet proinflammatory actions—contribute to thrombosis by (1) promoting chronic inflammation (via cytokines like interleukin-8 and others), (2) inducing endothelial changes (permeability, adhesion molecules), and (3) facilitating platelet and coagulation system activation. Their involvement demonstrates that not only overt immune activation but also tumor-induced immunosuppressive circuits, enhanced by ICI-driven feedback loops, play a role in ICI-associated thrombosis.

### 3.5. Endothelial and Platelet Activation

In patients receiving ICI therapy, who have a higher incidence of venous and arterial thrombotic events, cytokine profiles may serve as predictive markers. Clinical studies indicate that elevated neutrophil-to-lymphocyte ratios and increased cytokine levels correlate with greater thrombotic risk and poorer survival outcomes in these patients [45,46]. By blocking regulatory pathways, ICIs enhance immune responses, triggering systemic inflammation—a key driver of thrombosis [47,48].

Elevated levels of inflammatory cytokines, including interferon-gamma and tumor necrosis factor, are frequently observed in patients receiving ICI therapy [33]. These cytokines can directly activate endothelial cells, causing endothelial dysfunction. Activated endothelium upregulates adhesion molecules (e.g., E-selectin, vascular cell adhesion molecule 1) and procoagulant factors (e.g., TF) on its surface, which in turn enhances platelet adhesion and thrombin generation, thereby contributing to thrombosis [48,49]. For example, exposure to tumor necrosis factor has been shown to make endothelial cells highly adhesive to platelets and to increase platelet-dependent thrombin formation [49]. Thus, the inflammatory milieu fostered by ICIs translates into a prothrombotic state via endothelial activation.

Concurrent with endothelial changes, ICIs can also influence platelet function both indirectly (through inflammation) and directly. Inflammation leads to increased formation of NETs—web-like DNA structures expelled by activated neutrophils in response to factors like interferon-gamma. NETs serve as a scaffold for platelet adhesion and activation, and ICIs have been shown to induce NET formation as part of the heightened immune response. Indeed, T cell activation in ICI therapy can trigger neutrophils to release NETs [33], and elevated levels of circulating NETs have been detected in patients on ICIs [50]. Moreover, immunotherapy-treated patients show an increased presence of circulating neutrophil–platelet aggregates, which is a hallmark of platelet activation in the bloodstream. These aggregates arise when neutrophils (possibly through NET production) physically bind to platelets, and their increased frequency in ICI patients provides evidence of immune cell–platelet crosstalk, contributing to thrombus formation [33].

Beyond these indirect effects, ICIs may directly interact with platelets. Recent experimental studies have tested the direct impact of checkpoint inhibitor drugs on platelets in vitro. Interestingly, the results differ by drug: pembrolizumab (anti-PD-1) has been shown to stimulate platelet aggregation, whereas nivolumab (anti-PD-1) and ipilimumab (anti-CTLA-4) promote platelet disaggregation under the same testing conditions. Contrary to the anticipated reduction in thrombotic risk associated with attenuated platelet aggregation (observed with nivolumab and ipilimumab), data from the same study demonstrated that these agents paradoxically enhanced platelet procoagulant activity despite suppressing aggregation. Specifically, platelets exposed to nivolumab or ipilimumab showed enhanced thrombin generation capacity and a prolonged clot formation time with more stable clot architecture, indicating a net prothrombotic effect even in the absence of overt aggregation. Pembrolizumab, on the other hand, directly increased aggregation, which is a straightforward prothrombotic effect. These observations suggest that different ICIs influence platelet biology via distinct mechanisms: pembrolizumab causes platelets to stick together, whereas nivolumab and ipilimumab alter platelet function in subtler ways (perhaps through Fc receptors or signaling pathways), making them procoagulant without forming large aggregates. Importantly, all these effects can disrupt the balance between coagulation and fibrinolysis [51]. The combination of cytokine-mediated endothelial activation (promoting platelet adhesion) and mechanical forces (shear stress) on activated platelets further enhances platelet prothrombotic activity, illustrating the complex interplay underlying ICI-associated thrombosis [49,51]. In summary, platelet activation in ICI therapy is driven by inflammation (indirectly via cytokines and NETs) and can be modulated directly by the checkpoint inhibitors themselves. This dual impact ensures that platelets contribute significantly to the prothrombotic state in patients on ICIs.

### 3.6. Impaired Fibrinolysis

In vitro experiments have shown that T cell activation induces TF production in PD-L1-high monocytes, thereby promoting coagulation. This immune activation also suppresses fibrinolysis, likely through increased plasminogen activator inhibitor-1 (PAI-1) levels. These findings highlight the dual role of ICIs in driving thrombosis and emphasize the need to address this risk in cancer therapy. A retrospective analysis of patients with lung cancer treated with ICIs identified a link between immune activation and coagulation-fibrinolysis disorders. Tumor PD-L1 expression was associated with coagulation abnormalities and reduced fibrinolysis, suggesting its potential as a biomarker for thrombotic risk [34].

In parallel, immune activation has been shown to upregulate PAI-1, which plays a central role in suppressing fibrinolysis by inhibiting tissue-type and urokinase-type plasminogen activators. Beyond its classical role in the fibrinolytic cascade, PAI-1 also contributes to immune evasion through upregulation of PD-L1 on tumor cells and tumor-associated macrophages via the JAK/STAT pathway. This dual action—enhancing procoagulant potential while dampening antitumor immunity—highlights the multifaceted contribution of PAI-1 to the coagulation-immune axis in cancer. Notably, blockade of PAI-1 was shown to reduce PD-L1 expression, restore cytotoxic T cell activity, and promote tumor regression in preclinical models, underscoring its potential as a therapeutic target [52].

### 3.7. Cardiac Dysfunction

ICIs have been linked to adverse cardiovascular events, including myocarditis, heart failure, ischemic complications, and thrombosis. While they effectively enhance antitumor immunity, they can also trigger autoimmune responses against cardiac self-antigens [53,54], leading to sustained inflammatory cascades and tissue damage [55]. Furthermore, ICIs promote the upregulation of inflammatory mediators such as tumor necrosis factor, interferon-gamma, and interleukins, contributing to cardiac and endothelial dysfunction. These processes can destabilize atherosclerotic plaques and promote thrombosis, further increasing the risk of cardiovascular complications [53,55].

### 3.8. Exacerbating Factors

Patients with a history of thrombotic events have a significantly higher risk of recurrence when treated with ICIs, with studies suggesting up to a threefold increase compared to those without prior thrombosis [56]. Obesity also contributes to thrombotic risk, as higher body mass index is associated with increased event rates [57,58]. Conversely, the influence of age remains uncertain. Some studies report no significant increase in thrombotic risk among patients aged 65 or older [56], whereas others suggest that younger individuals may be at greater risk [59,60]. Smoking is another well-established risk factor, substantially increasing thrombotic risk in ICI-treated patients [56].

Beyond patient-specific factors, several comorbid conditions further elevate thrombosis risk in ICI-treated patients. Dyslipidemia significantly increases the likelihood of thrombotic events [61], and chronic obstructive pulmonary disease has also been linked to a higher risk [56]. Immune-related adverse events contribute as well, with studies reporting a more than twofold increase in VTE risk [62]. Hypertension has been associated with a 37% higher VTE risk and is part of a broader set of risk factors that collectively double the thrombotic risk in ICI-treated patients compared with non-ICI groups [60,63].

In addition to patient-specific and comorbid risk factors, treatment-related variables also influence thrombotic risk in ICI-treated patients. Combination therapy with ICIs significantly increases the likelihood of thrombosis compared with monotherapy or other treatment regimens. Le Sève et al. reported a one-year cumulative VTE incidence of 29.3% for nivolumab and ipilimumab combination therapy, compared with 9.1% for nivolumab alone and 14.9% for pembrolizumab alone [62]. Van Dorst et al. further highlighted that nivolumab and ipilimumab combination therapy substantially increases thrombotic risk, with a sub-hazard ratio of 2.5 [57]. Similarly, Wang and Carrier found that combining ICIs with chemotherapy or using multiple ICIs together may elevate VTE risk compared with single-agent therapies. Connors et al. identified ipilimumab as the ICI with the highest VTE risk compared with pembrolizumab, whereas durvalumab had the lowest risk [58,59].

The initiation of anticoagulation therapy at or after starting ICI treatment—when not specifically for VTE treatment—has been associated with a significant reduction in VTE risk. This suggests a potential benefit of prophylactic anticoagulation in patients with multiple risk factors undergoing ICI therapy. Specifically, initiating anticoagulation was linked to an approximately 40% lower hazard of VTE compared with those who had not yet started or never received anticoagulation [58].

Although the factors discussed above are significant, a broader context must be considered. The hypercoagulable state associated with cancer, combined with the prothrombotic effects of ICIs, highlights the complexity of managing thrombotic risk in this population. Moreover, the potential for ICIs to exacerbate atherosclerotic lesions and the increased risk in patients with atrial fibrillation or ischemic heart disease suggest the need for careful monitoring and, in high-risk cases, consideration of prophylactic anticoagulation [59,63].

Table 1 provides a summary of the key molecules and cellular players identified in ICI-associated thrombosis, along with their roles and the context in which they have been studied.

## 4. Reported ICI-Associated Thrombosis in the Literature

A retrospective, single-center cohort study at Massachusetts General Hospital evaluated the correlation between ICIs and VTE risk. The study analyzed medical records of 2854 patients who received ICIs, with a median follow-up of 194 days. Table 2 summarizes patient demographics and common cancer types. Notably, PD-1 inhibitors were the most prescribed, with 75.2% of patients receiving immunotherapy. The study found that the incidence of both pulmonary embolism (PE) and deep venous thrombosis (DVT) increased over time following ICI initiation. VTE risk peaked early after starting ICI therapy and remained elevated compared with pre-ICI levels, despite declining after the initial treatment phase. At six months and one year post-ICI, the VTE incidence was 7.4% and 13.8%, respectively, with a fourfold increase in DVT or PE risk. Younger patients had higher VTE rates two years after starting ICIs (mean age: 63 ± 12 years) compared with VTE-free patients (mean age: 65 ± 13 years). Furthermore, univariate and multivariate analyses showed that patients with VTE had higher Khorana scores (KS)—a key tool for estimating VTE risk—with a score above 2 significantly associated with increased risk of ICI-associated VTE. VTE incidence increased across all cancer groups at two years, although melanoma patients had a lower risk compared with lung cancer patients. Finally, no significant difference was observed between ICI classes or with prior cardiotoxic chemotherapy exposure [60].

Findings from a retrospective study at Shinshu University Hospital further support the link between ICIs and thrombotic risk. Among 548 ICI-treated patients followed for a mean of 15.1 months, 6.9% developed thrombosis, with VTE and arterial thrombotic events occurring in 4.0% and 2.9% of cases, respectively. A significant correlation was observed between lipid abnormalities and post-ICI thrombosis. Consistent with previous findings, a KS above 2 and each one-point increase in the score were significantly associated with a higher risk of VTE or arterial thrombosis. In addition, urothelial cancer was identified as a significant risk factor for thrombotic events [61].

Further supporting the association between ICIs and thrombosis, a study by the Saudi Food and Drug Authority analyzed individual safety case reports from the World Health Organization Program for International Drug Monitoring. Screening data from 1976 to 2020, the study identified 161 patients on anti-PD-1 or anti-PD-L1 therapy who developed VTE or arterial thrombosis. VTE was more common than arterial events, with an incidence of 51.6%, followed by myocardial infarction (24.8%), PE (6.8%), and acute coronary syndrome (5.0%). Notably, older age was a contributing factor, as the median age was 68 years, suggesting an increased thrombotic risk in patients over 65 years of age [65].

Contrary to previous findings, a retrospective study in China suggested a potential protective effect of ICIs against VTE in patients with non-small cell lung cancer (NSCLC). Among 730 patients reviewed between 2019 and 2021, only 19 of 166 ICI-treated patients developed VTE, with sintilimab being the most commonly used agent. Interestingly, a multivariate analysis showed that ICI use was associated with a reduced VTE risk. The authors hypothesized that ICIs may lower tumor burden, thereby mitigating the preexisting hypercoagulable state. However, univariate and multivariate analyses confirmed that advanced clinical stage (III–IV) and a KS of 2 or higher remained independent risk factors for VTE in patients with NSCLC [64].

A cohort study from the United States analyzed data from the National Veterans Affairs Database to compare VTE risk in ICI-treated and chemotherapy-treated patients. The study included 1823 patients in the ICI group and 6345 in the chemotherapy group. At six months, VTE incidence was similar between groups (7.71% for ICIs vs. 7.54% for chemotherapy). Lung cancer had the highest VTE incidence among ICI-treated patients (9.55% for NSCLC and 8.47% for small cell lung cancer), whereas melanoma had the lowest (3.63%). Patients receiving only ICIs had the highest six-month VTE rate (10.4%), compared with 9.6% for the ICI/chemotherapy group and 8.91% for chemotherapy alone. The study could not identify specific VTE risk factors owing to demographic limitations—most patients were male—and the inclusion of only advanced cancer stages. The authors concluded that ICI-associated VTE rates are comparable to those of traditional chemotherapy, suggesting ICIs should be classified in the same thrombotic risk category as chemotherapy [66].

Expanding on ICI-associated thrombosis in specific cancer types, a multicenter cohort study by the Spanish Society of Medical Oncology Thrombosis and Cancer Group assessed VTE and arterial thrombosis risk in patients with head and neck cancer receiving ICIs. Conducted across nine centers, the study included 143 patients diagnosed between 2015 and 2021, with a median follow-up of 8.6 months. The overall incidence of VTE or arterial thrombosis was 2.8%, occurring in patients who received at least 2.5 cycles of ICIs. Thrombotic events did not impact overall survival, but liver metastases were identified as a predictive factor for VTE and arterial thrombosis. The authors suggested that alcohol use may have been a confounding factor, potentially contributing to liver and platelet damage, thereby increasing thromboembolic risk [67].

Building on the cardiovascular risks associated with ICIs, a large cohort study at Massachusetts General Hospital evaluated the association between ICI use and atherosclerotic cardiovascular events. This single-center study employed two designs: a matched cohort analysis comparing 2842 ICI-treated patients to 2842 non-ICI-treated patients matched by age, cancer type, and cardiovascular history (2008–2012) and a case-crossover analysis assessing cardiovascular event rates before and after ICI initiation. NSCLC and melanoma were the most common cancers, with anti-PD-1 therapy being the most frequently prescribed. ICI use was associated with more than a fourfold increase in cardiovascular events and a three- to fourfold rise in coronary revascularization and ischemic stroke. In the case-crossover analysis, 119 ICI-treated patients experienced cardiovascular events in the two years post-ICI, compared with 66 events in the two years before treatment, translating to an event rate increase from 1.37 to 6.55 per 100 person-years. CT scans revealed significant plaque progression over three imaging time points, with post-ICI plaque growth occurring at a rate three times higher than in patients with clinical or subclinical cardiovascular disease. These findings further support the hypothesis that ICIs accelerate atherosclerosis and increase thrombotic risk [68].

The available literature on ICI-associated thrombosis is based on retrospective chart reviews, which may be a limitation. Studies included provide the most comprehensive review of incidence and risk factors of ICI-associated thrombosis. As a result, prospective studies are needed to better characterize the relationship between ICI initiation and thrombotic events while controlling for confounding factors such as cancer stage, comorbidities, and concurrent therapies.

## 5. Management of ICI-Associated Thrombosis

The management of ICI-associated thrombosis is similar to that of other types of cancer-associated thrombosis and involves anticoagulation with agents such as low-molecular-weight heparin (LMWH) and direct oral anticoagulants (DOACs), which remain the cornerstone of thrombosis management in patients with cancer. The choice of anticoagulant depends on various factors, including underlying risk factors, tumor characteristics, bleeding risk, patient adherence and follow-up, cost-effectiveness, and potential drug–drug interaction [69,70].

A randomized clinical trial compared long-term tinzaparin with warfarin for the treatment of acute VTE in patients with cancer. The trial demonstrated that tinzaparin was more effective than warfarin for the secondary prevention of VTE without a significantly increased risk of bleeding. In addition, tinzaparin was associated with a lower risk of post-thrombotic syndrome compared with warfarin [71]. In patients with cancer and VTE, LMWH is associated with a lower risk of recurrent VTE without a significant increase in major bleeding complications compared with vitamin K antagonists (VKAs) [72]. Similarly, another trial, which compared LMWH (enoxaparin) with warfarin for cancer-associated VTE, revealed that LMWH was superior in preventing thrombosis recurrence and was associated with fewer bleeding complications [73]. The superiority of LMWHs over VKAs is attributed to fewer drug–drug interactions and reliable anticoagulant effects via parenteral administration. However, the need for daily injections and associated costs pose practical and financial challenges, often leading to non-adherence and a greater likelihood of switching to oral anticoagulants compared with VKAs.

DOACs, including three direct factor Xa inhibitors (apixaban, edoxaban, and rivaroxaban) and one direct thrombin inhibitor (dabigatran), are currently indicated for the acute treatment of VTE. However, caution is advised in patients with upper gastrointestinal cancers or unresected luminal tumors owing to an increased bleeding risk. According to the Hokusai VTE Cancer trial, edoxaban was associated with a lower rate of recurrent VTE but a higher rate of bleeding compared with the LMWH dalteparin [74]. Similarly, the SELECT-D trial found that rivaroxaban resulted in relatively low VTE recurrence but a higher incidence of clinically relevant non-major bleeding compared with dalteparin in patients with cancer and VTE [75]. These trials suggest that although DOACs may be effective for VTE management, they are associated with a higher bleeding risk compared with LMWH.

Data on the optimal treatment duration for ICI-associated thrombosis remain limited. However, guidance can be extrapolated from cancer-associated thrombosis data. Balancing the risks (e.g., bleeding) and benefits (e.g., preventing recurrent thrombosis) is essential when determining treatment duration. Current guidelines for the acute and long-term management of VTE to prevent recurrence suggest initial anticoagulation with LMWH, unfractionated heparin (UFH), fondaparinux, or rivaroxaban. According to the 2023 update from the American Society of Clinical Oncology, LMWH is preferred over UFH for the first 5–10 days of anticoagulation in patients with cancer and newly diagnosed VTE, provided there is no severe renal impairment (creatinine clearance < 30 mL/min). If LMWH or DOACs are not accessible, VKAs are an alternative.

During the first six months, LMWH, edoxaban, or rivaroxaban are favored over VKAs owing to improved efficacy. However, the elevated bleeding risk associated with DOACs, particularly in patients with gastrointestinal or potentially genitourinary malignancies, must be considered. Drug–drug interactions should also be assessed before initiating DOACs [76]. For long-term anticoagulation, the American Society of Hematology recommends continuing anticoagulation for secondary prophylaxis beyond 6 months in patients with active cancer and VTE, rather than limiting treatment to 3–6 months. After the initial six-month period, anticoagulation with LMWH, DOACs, or VKAs should be evaluated on a case-by-case basis. For instance, patients with active cancer—such as those with metastatic disease or undergoing chemotherapy—may benefit from extended anticoagulation. The American Society of Hematology guideline panel suggests using either DOACs or LMWH for long-term anticoagulation [69].

The role of antiplatelet agents, particularly in distinguishing their impact on arterial versus venous thrombosis in patients treated with ICIs, is an important area for discussion. Two meta-analyses demonstrated that combined therapy with antiplatelet agents (such as aspirin) and anticoagulants significantly reduced the risk of VTE recurrence in patients with cancer [77,78]. However, data on optimal treatment strategies for ICI-associated arterial thrombosis remain limited. According to guidelines for cerebrovascular disease and stroke, standard-of-care approaches similar to those used in the non-cancer population are generally followed. These include the use of antiplatelet agents with or without anticoagulation, management of cardiovascular risk factors (e.g., controlling blood pressure and diabetes, smoking cessation), and revascularization when indicated [9,79].

Nevertheless, all antiplatelet agents carry a notable bleeding risk, which is associated with poor prognosis and high mortality [80,81]. For example, one study showed that 100 mg/day of aspirin reduced vascular events by 12% but increased the risk of major bleeding by 29% [82]. Current antiplatelet agents, such as P2Y12 receptor antagonists (clopidogrel, prasugrel, and ticagrelor), thrombin-activated receptor-1 antagonists, and αIIbβ3 inhibitors (abciximab, eptifibatide, and tirofiban), exhibit strong antithrombotic effects but are limited in clinical use due to bleeding risks [83]. This highlights the need for new antithrombotic therapeutic targets. A recent animal model study demonstrated that targeting glycoprotein VI and integrin α6 reduced thrombosis without causing severe bleeding [84]. Moreover, targeting CD39 or modulating αIIbβ3 to inhibit outside-in signaling offers promising strategies for reducing thrombosis while minimizing bleeding risk [85].

It is important to acknowledge that current management strategies for thrombosis in patients receiving ICIs are largely extrapolated from existing guidelines for cancer-associated thrombosis. However, these guidelines carry inherent limitations, as they do not specifically address the unique thrombotic mechanisms potentially associated with ICIs. In the absence of robust, ICI-focused data, such extrapolation may be clinically insufficient, highlighting the urgent need for dedicated studies and tailored recommendations.

## 6. Prevention of ICI-Associated Thrombosis

Risk stratification is crucial in determining whether to use anticoagulation for primary prevention in patients with cancer. This decision should consider not only the risk of VTE and anticoagulation-associated bleeding but also the cost and mode of anticoagulant administration, both of which may affect quality of life. Patients are generally stratified based on whether they are in a hospitalized or ambulatory setting [86]. Hospitalized patients are further categorized according to whether they are admitted for an acute illness or surgical procedures. Notably, patients with cancer are at a particularly high risk for VTE across all these settings. For example, patients with cancer admitted for medical illness have a higher risk of VTE compared with other inpatient populations [87]. However, the benefits of thromboprophylaxis remain poorly evaluated in certain subgroups, particularly patients with hematologic malignancies, in view of common abnormalities in hematologic parameters [88].

Although anticoagulation has been proven to reduce the risk of VTE in patients with cancer, evidence of its impact on mortality remains lacking. Moreover, some patients with cancer, particularly those with hematologic malignancies or receiving myelosuppressive therapies, have a higher risk of bleeding [87]. Unfortunately, these patients have often been excluded from clinical trials investigating primary thromboprophylaxis [88]. Therefore, individualized VTE risk assessment and careful evaluation of the risk–benefit balance are essential when considering VTE prophylaxis in patients with cancer [86].

The risk of VTE is not equally distributed among patients with cancer but varies considerably depending on the presence of specific risk factors. This underscores the need for individualized assessment using risk scores to identify patients who would benefit most from thromboprophylaxis [89]. In addition, phenomena such as recurrent VTE or de novo VTE despite appropriate thromboprophylaxis or therapeutic anticoagulation remain poorly understood, with implicated risk factors yet to be confirmed [90]. The literature on risk factors for VTE development in cancer is extensive. Non-modifiable risk factors include patient-related factors such as older age, inherited coagulopathies (e.g., thrombophilia), and non-O blood groups. A recent meta-analysis confirmed the role of inherited risk factors in VTE development across various age groups and cancer types. For instance, women with early-stage breast cancer and the Factor V Leiden mutation who received tamoxifen endocrine therapy had significantly higher odds of developing VTE. Furthermore, 46% of children with acute lymphoblastic leukemia and a prothrombotic mutation, such as MTHFR C677T, developed thrombosis compared with only 2.2% in the control group [91]. Studies have also shown that certain abnormal coagulation profiles, such as elevated D-dimer and fibrinogen levels, could help identify patients with cancer who are at higher risk for VTE occurrence.

Potentially modifiable risk factors for VTE include intra-abdominal procedures, infections, and short life expectancy. Tumor-specific factors also play a role, such as tumor cells expressing procoagulant factors that trigger thrombin formation or anatomical factors such as the compression of large vessels, as seen in hepatocellular carcinoma, which can lead to VTE. Moreover, some cancers, such as renal cell carcinoma, may infiltrate vessels such as the inferior vena cava, causing obstruction and subsequent thrombosis in up to 9% of patients [92]. Importantly, studies have shown that the type of cancer is the most significant risk factor for VTE in patients with cancer. Cancers such as pancreatic, ovarian, and lung cancers are associated with a particularly high risk of thrombosis, contributing to the asymmetric distribution of VTE risk among patients [86].

Patients with cancer undergoing active treatment with chemotherapy or targeted therapy are at an increased risk of developing VTE [86]. Recent post-marketing surveillance for ICIs has also revealed a significant risk of thrombotic events, including arterial thrombosis and VTE [86]. This finding is particularly relevant given the survival benefits associated with ICIs and their growing use in the treatment of various hematologic and oncologic cancers. The risk of VTE with ICI use appears to be highest during the initial phase of treatment and gradually decreases over time, suggesting a potential induction phase risk or a physiological adaptation mechanism. Notably, risk factors for VTE in the context of ICI use differ from those typically observed. For instance, the risk of VTE is higher with dual-ICI therapy compared to single-agent therapy. In one cohort study, younger age (≤65 years) and increased expression of PD-L1 on tumor cells were associated with higher odds of VTE. Another large retrospective cohort study found that metastatic disease and younger age at diagnosis were linked to both an increased risk of VTE and decreased survival in patients who developed VTE [48]. Across all available studies, sex and ethnicity were not associated with an increased risk of VTE in patients with cancer receiving ICIs. Despite the evidence supporting an association between ICI use and VTE risk, causality remains unproven. The observed association may be confounded by the duration of ICI exposure [44].

Given the multifactorial nature of VTE risk in patients with cancer—particularly those receiving ICI therapy—the decision to offer primary VTE prophylaxis depends on appropriate risk stratification. The KS is the most widely used risk assessment tool for guiding thromboprophylaxis in patients with cancer [86,88,93]. Recent validation studies have lowered the cutoff for high-risk patients to a KS of 2 or more, thereby expanding the population eligible for primary VTE prophylaxis. Some studies have also suggested that incorporating additional variables, such as D-dimer levels, into the KS formula may enhance its predictive accuracy, highlighting the importance of individualized risk assessment. However, other investigations attempting to validate the KS in different oncologic populations have shown that it fails to identify a substantial proportion of at-risk patients, particularly those with hematologic malignancies such as multiple myeloma. To address this, current studies are evaluating alternative tools such as the SAVED and IMPEDE scores to improve VTE risk prediction in patients who may be missed by the KS [94,95,96]. On a relevant note, the KS was recently evaluated for its validity in estimating the risk of VTE associated with ICI use in comparison with the Padua and Caprini scores [97]. Although the KS showed poorer discriminative ability across the risk strata, it is important to note that the comparisons differed among the scores: for the KS, the comparison was between moderate- and high-risk groups; for the Padua score, it was high versus low; and for the Caprini score, it was low versus moderate versus high. Notably, the Caprini score also showed diminished discriminative ability between its low- and moderate-risk categories. This highlights our previous understanding that the ability of a risk score to discriminate between adjacent strata might not be absolute given that patients’ risk factors may not always be clearly defined.

On the other hand, we acknowledge that other risk scores, such as RAM, SAVED, and IMPEDE, have not been validated in the context of ICI-associated thrombotic risk. Nonetheless, several published cohorts have used some of these risk scores to assess thrombotic risk in patients receiving ICIs and have described how the results were used to guide thrombotic risk and anticoagulation use [58,95,96,98]. The evidence gap in having a validated risk assessment score for ICI-associated thrombosis could lay the grounds for future investigations in such scores for patients on ICIs.

In hospitalized patients, recent decision analysis models have been developed to assess both thrombosis and bleeding risks. One such model utilizes the IMPROVE VTE score and IMPROVE Bleeding score, applying a threshold of ≥2 for VTE risk and ≤7 for bleeding risk to identify patients eligible for thromboprophylaxis with minimal bleeding complications [99,100]. Another recently developed model is the ONCOTHROMB score, which incorporates genetic risk factors and refines the KS by stratifying tumor type and body mass index using TNM staging to enhance VTE risk prediction precision [101]. In the context of ICI-based therapy, a recent large cohort study validated the utility of the KS for risk stratification in patients receiving ICIs. The study reported a 4.1% risk of thromboembolic events in patients with a KS of ≥2, suggesting eligibility for primary thromboprophylaxis. The study also showed that the six-month risk of VTE and arterial thrombosis paralleled the KS risk strata, with rates of 2.1% for low-risk, 2.6% for intermediate-risk, and 3.7% for high-risk patients [102].

Data on VTE prophylaxis in patients with cancer remain limited and controversial. A meta-analysis found that, in sensitivity analyses for cancer subpopulations, the relative risk for thromboembolic events among hospitalized patients with cancer receiving thromboprophylaxis versus placebo was not significantly different [88]. Similarly, Spyropoulos et al. reported that VTE prophylaxis in patients with cancer reduced the risk of PE but did not significantly reduce the risk of symptomatic proximal or distal DVT or in-hospital mortality. The authors suggested that the limited impact of thromboprophylaxis observed in studies could be attributed to the rarity of VTE events, the occurrence of VTE post-discharge, and the frequent asymptomatic nature of these events, which may lead to under-detection [103]. Given these uncertainties, decisions regarding thromboprophylaxis in patients with cancer—particularly those receiving ICIs—should be guided by case-specific scenarios, relevant studies, or extrapolation from general population trials involving patients with cancer who do not conform to current evidence [104]. Historical trials, such as PREVENT and ARTEMIS, along with large meta-analyses, have demonstrated the effectiveness of thromboprophylaxis in preventing VTE among high-risk inpatients, including those on ICIs. However, further studies are needed to assess VTE risk following the discontinuation of thromboprophylaxis [105].

Guidance from international hematology and oncology societies, such as the American Society of Clinical Oncology, American Society of Hematology, and International Initiative on Thrombosis and Cancer, also informs thromboprophylaxis recommendations based on clinical scenarios and risk stratification. For example, the 2019 American Society of Clinical Oncology guidelines recommend thromboprophylaxis for all hospitalized patients with active malignancy and reduced mobility but not for those admitted for minor procedures or chemotherapy. In contrast, the American Society of Hematology guidelines recommend thromboprophylaxis for all hospitalized patients with active malignancy [86]. Moreover, the International Society on Thrombosis and Haemostasis recommends LMWH for patients with acute lymphoblastic leukemia receiving L-asparaginase owing to its high associated VTE risk. Regarding patients on ICIs, available data are limited to retrospective cohort studies, with findings varying based on cancer type and other risk factors. Nevertheless, evidence suggests that prolonged ICI exposure increases the risk of developing VTE and/or arterial thrombosis. Currently, the KS remains the only validated tool to guide risk stratification and initiation of thromboprophylaxis in these patients, similar to its use in patients receiving chemotherapy [59].

International guidelines for thromboprophylaxis in patients with active cancer continue to recommend heparin-based prophylaxis with either LMWH or UFH in the inpatient setting. However, recent randomized controlled trials, including AVERT and CASSINI, have demonstrated that prophylactic DOACs, specifically apixaban and rivaroxaban, are effective and safe in preventing cancer-associated thromboembolism among ambulatory patients at intermediate to high risk [106,107,108]. Currently, no head-to-head trials have compared LMWH/UFH to DOACs for thromboprophylaxis in patients with cancer receiving chemotherapy. However, a meta-analysis showed that both LMWH and DOACs offer comparable thromboprophylactic effects [106]. Conversely, a meta-analysis of the MAGELLAN, ADOPT, and PEX trials found that DOACs were not superior to LMWH in the inpatient setting and were associated with an increased risk of major bleeding [106].

Preclinical studies investigating resistance mechanisms to ICIs have implicated factor X in immune escape mechanisms, suggesting a potential role for factor Xa inhibitors such as fondaparinux in enhancing the anti-tumor effects of ICIs [109,110]. However, robust clinical studies are needed to validate the theoretical anti-tumoral synergy between anticoagulants and ICIs, whether in prophylactic or therapeutic settings. For patients with cancer receiving ICIs, the choice of prophylactic anticoagulant aligns with general recommendations for cancer-associated VTE prophylaxis. Either LMWH or DOACs can be used, with the choice tailored to patient and tumor characteristics, cost considerations, and patient preference [69,70]. Regarding the duration of prophylaxis, evidence suggests that thromboprophylaxis should be discontinued upon hospital discharge or once the patient is fully ambulatory. A meta-analysis comparing extended-duration to standard-duration thromboprophylaxis demonstrated no additional benefit in reducing short-term or long-term thromboembolic risk with extended prophylaxis [111].

## 7. Future Directions

As ICIs continue to transform cancer treatment, the complexity of their adverse effects, including VTE, and the variability in treatment response highlight the need for further research in several key areas. A critical focus is understanding the mechanisms underlying immune-related thrombotic events. Studies indicate that ICI-induced inflammation, reflected in early changes in C-reactive protein levels, correlates with an increased VTE risk, suggesting that C-reactive protein trajectories may serve as a biomarker for predicting thrombotic complications during ICI therapy [112]. Moreover, emerging evidence suggests that immune-related adverse events, including cardiovascular toxicities such as thrombosis, may be associated with specific biomarkers, such as the neutrophil-to-lymphocyte ratio, baseline circulating tumor cell levels, and post-treatment lactate dehydrogenase levels, which could help identify patients at risk of severe complications [113].

Mechanistic research on immune response pathways underscores the critical role of immune cell communication in determining ICI efficacy. A network-based machine learning approach analyzing patient-specific cell communication networks has shown that certain communication pathways can accurately predict patient responses to ICIs [114]. This method could be further refined to improve patient selection and identify high-risk individuals, ultimately enhancing clinical outcomes. The integration of artificial intelligence (AI) into ICI research is also advancing, offering new opportunities to enhance precision oncology. AI models can predict patient responses, optimize combination therapy strategies, and manage immune-related adverse events by analyzing large datasets, including genomic and imaging data [115]. AI-driven biomarker discovery is particularly promising for identifying patterns in immune-related toxicity profiles, which could guide personalized treatment adjustments to minimize adverse effects [113]. Continued investment in AI-driven research and global collaborations will be essential to fully realize this potential.

Future studies should prioritize the identification of predictive biomarkers for ICI therapy. While tumor mutational burden and PD-L1 expression remain standard markers, emerging biomarkers—such as circulating tumor DNA, gut microbiota composition, and immune gene signatures—are showing promise in predicting treatment response and adverse events [116,117]. Notably, recent studies suggest that patients who develop immune-related adverse events tend to experience improved treatment outcomes, highlighting the need for biomarkers that can predict both efficacy and toxicity [113]. Developing comprehensive predictive models that integrate multiple biomarkers is expected to significantly advance precision immuno-oncology [118].

Addressing the intricate link between ICI response and thrombotic risk requires further investigation. Predictive biomarkers of robust anti-tumor response, often associated with heightened inflammatory states, might paradoxically signal an increased susceptibility to thrombotic events [112]. Understanding this connection could refine risk stratification, allowing for targeted thromboprophylaxis in predicted responders who may be at higher thrombotic risk, thereby improving the overall management of these patients. While several retrospective studies and meta-analyses have begun to quantify ICI-associated thrombotic risk, significant heterogeneity exists in study populations, ICI regimens, and endpoint definitions [59,119,120]. Future meta-analyses incorporating standardized data collection from prospective trials or large real-world evidence registries will be crucial for a more definitive understanding. Furthermore, AI offers a pathway to overcome some current data limitations. Although large datasets focused specifically on ICI-associated thrombosis are still evolving, AI models can integrate multi-modal data—including clinical variables, existing biomarkers (such as D-dimer and C-reactive protein), genomic data, imaging features, and ICI response indicators—from broader cancer and thrombosis datasets [121]. Such models could identify complex, non-linear patterns predictive of thrombotic risk in the specific context of ICI therapy, potentially improving upon traditional risk scores [122] and enabling more personalized prevention strategies even before large, dedicated datasets become available.

Combination therapies represent a promising strategy for enhancing ICI outcomes. Integrating ICIs with chemotherapy, radiation therapy, or novel immune-targeting agents has demonstrated synergistic effects in clinical trials [123]. These approaches may help overcome resistance mechanisms and improve efficacy in patients who do not respond to monotherapy. However, the impact of combination therapies on thrombotic risk remains underexplored. A recent Bayesian network meta-analysis identified significant differences in thromboembolic event risks among various ICI regimens, with ipilimumab associated with the highest risk of VTE. These findings underscore the need for personalized thromboprophylaxis strategies tailored to specific treatment regimens [119].

The association between immune-related VTE and treatment outcomes underscores the need for tailored risk management strategies. Recent meta-analyses showed that patients who develop VTE during ICI therapy experience worse overall survival, highlighting the importance of early identification and management of thrombotic risks [119,120]. Future clinical trials should focus on establishing optimal anticoagulation protocols for these patients while carefully balancing the risk of bleeding. In addition, research should aim to elucidate the mechanisms underlying ICI-associated complications, refine predictive models using AI and novel biomarkers, and optimize combination therapy strategies. These efforts will advance more personalized, effective, and safer cancer immunotherapy.

## 8. Conclusions

ICIs have transformed cancer treatment, but their association with thrombotic complications remains a significant concern. ICI-associated thrombosis arises from multiple mechanisms, including immune dysregulation, T cell activation, endothelial dysfunction, increased TF expression, and impaired fibrinolysis. Patient-specific factors such as obesity, smoking, and prior thrombotic events, along with treatment-related factors such as combination therapy and higher KS, further heighten this risk. Notably, thrombotic events during ICI therapy are associated with poorer survival outcomes, highlighting the need for proactive risk assessment and management.

Current treatment follows standard protocols for cancer-associated thrombosis, primarily using LMWH or DOACs. However, the optimal treatment duration and the role of prophylactic anticoagulation in high-risk patients receiving ICI therapy require further investigation. Given the distinct mechanisms and timing of ICI-associated thrombosis, tailored prophylactic strategies are essential, with the KS serving as a valuable tool for risk stratification.

Future research should prioritize identifying predictive biomarkers, optimizing anticoagulation strategies, and exploring potential synergies between anticoagulants and ICIs to improve therapeutic outcomes. Prospective studies are essential to develop evidence-based guidelines for managing thrombotic risk in patients receiving ICI therapy, ensuring effective cancer treatment while minimizing thrombotic complications.

## Figures and Tables

**Table 1:** Key molecules and factors involved in ICI-associated thrombosis.

Molecule/Factor	Role in ICI-Associated Thrombosis
PD-1/PD-L1 checkpoint	Checkpoint molecules that normally dampen T cell activation; ICI blockade of PD-1/PD-L1 leads to hyperactive T cells and excessive inflammation, lowering self-tolerance and triggering autoimmunity (creating a thrombosis-prone state) [27,28,29,30,31].
Tumor necrosis factor	Proinflammatory cytokine elevated during ICI-induced immune responses; directly activates endothelial cells (upregulating adhesion molecules and tissue factor) and promotes a procoagulant state (also increases PAI-1 release, tipping the hemostatic balance toward thrombosis) [33,48].
Interferon-gamma	Th1 cytokine released by activated T cells (enhanced by PD-1 blockade); induces tissue factor expression on tumor cells and monocytes, linking immune activation to coagulation activation and thrombin generation [33,34].
Interleukin-8	Inflammatory chemokine often produced by MDSCs and other myeloid cells; high interleukin-8 levels correlate with increased risk of ICI-associated VTE. Interleukin-8 recruits neutrophils and can stimulate NET formation, providing a bridge between inflammation and thrombosis [44].
Tissue factor	Key initiator of the extrinsic coagulation cascade; upregulated on tumor cells and monocytes in ICI therapy via interferon-gamma from activated T cells [33,34]. Elevated TF leads to increased thrombin generation and fibrin clot formation, markedly heightening thrombotic risk [33,34,35].
Plasminogen activator inhibitor-1	Inhibits fibrinolysis by blocking tissue- and urokinase-type plasminogen activators; upregulated by immune activation during ICI therapy. Also promotes PD-L1 expression via JAK/STAT, linking thrombosis and immune evasion [52].
Myeloid-derived suppressor cells	Immunosuppressive myeloid cells that expand during cancer and can increase further with ICI therapy. High MDSC levels are associated with higher VTE incidence in ICI-treated patients [44]. MDSCs secrete prothrombotic inflammatory mediators (e.g., interleukin-8) and promote vascular permeability and aberrant angiogenesis [39,40,41], creating a thrombogenic microenvironment despite their immunosuppressive label.
Neutrophil extracellular traps and neutrophil–platelet aggregates	NETs are web-like DNA/protein networks extruded by activated neutrophils that trap platelets and red cells, promoting coagulation. ICI-induced inflammation (via T cells and cytokines) increases NET formation. Neutrophil–platelet aggregates (complexes indicating platelet activation by neutrophils) are found at higher levels in patients on ICIs, highlighting immune cell–platelet crosstalk in thrombosis [33].
Platelet activation/aggregation	Platelets are central to thrombosis; their activation is enhanced by cytokine-activated endothelium and NETs in ICI-treated patients. ICIs can also directly affect platelets: e.g., pembrolizumab increases platelet aggregation, whereas nivolumab and ipilimumab reduce aggregation but paradoxically enhance platelet procoagulant activity [33,51]. Overall, ICIs drive platelets toward a prothrombotic phenotype.
Antiphospholipid antibodies	Autoantibodies (e.g., anticardiolipin, anti-β2 glycoprotein I) that cause thrombosis in antiphospholipid syndrome. ICI therapy has been linked to the new onset of antiphospholipid antibodies in some patients, including cases of catastrophic antiphospholipid syndrome [25,26]. These antibodies can mediate widespread thromboses, illustrating a direct autoimmune mechanism of ICI-related clotting.
Soluble VCAM-1	A soluble form of an endothelial adhesion molecule, released during endothelial activation or damage. Elevated sVCAM-1 was identified as a predictor of thromboembolism in patients receiving ICIs (reflecting endothelial inflammation/activation) [44]. It is a biomarker linking immune activation to thrombosis risk.

**Table 2:** Summary of risk factors and incidence of thrombosis in ICI-treated patients with cancer. This table summarizes multiple studies on risk factors for thrombosis, specifically focusing on the incidence of arterial thromboembolism (ATE) and venous thromboembolism (VTE) in patients with cancer. It presents risk factors, ATE and VTE incidence rates, cancer types and stages, patient demographics (sex and median age), sample sizes, study designs, and study titles or descriptions.

Study	Study Design	Sample Size	Median Age (Years)	Sex (M/F)	Cancer Stage	Cancer Type	VTE Incidence (%)	ATE Incidence (%)	Risk Factors for Thrombosis
Immune checkpoint inhibitors for cancer and venous thromboembolic events [60]	Single-center retrospective chart review	2854	64 ± 13 years old	Male: 1640 Female: 1214	-	NSCLC (28.4%) Melanoma (28.2%)	7.4% at 6 months 13.8% at 1 year	-	Hypertension High Khorana risk score Young age
Thromboembolism during immune checkpoint inhibitor therapy: frequency and risk factors [61]	Single-center retrospective chart review	548	70.0	Male: 391 Female: 157	-	NSCLC (36.1%) Melanoma (19.9%)	4.0%	2.9%	Metabolic lipid abnormalities High Khorana risk score
Evaluating the effect of immune checkpoint inhibitors on venous thromboembolism in patients with non-small cell lung cancer [64]	Single-center retrospective chart review	730	336 (46.0%) ≥ 65 years old 394 (54%) < 65 years old	Male: 475 Female: 255	-	NSCLC (100%)	11.4%	-	Advanced stage High Khorana risk score
Immune checkpoint inhibitors and potential risk of thromboembolic events: analysis of the WHO global database of individual case safety reports [65]	Retrospective chart review of individual safety case reports	161	68	Male: 102 Female: 59	-	Lung Cancer (52.8%) RCC (149.9%) Melanoma (12.4%)	PE (51.6%) DVT (9.9%) DVT and PE (6.8%)	MI (24.8%) ACS (5.0%) Embolic Stroke (1.9%)	Age (>65) Male sex
Venous thromboembolism risk in patients with cancer receiving first-line immune checkpoint inhibitor versus chemotherapy [66]	Single center retrospective chart review	1823 (ICI group) vs. 6345 (chemo group)	69.4 (ICI group) vs. 67.8 (chemo group)	Male: 96%	III–IV	Lung cancer Kidney cancer Melanoma	7.71%	-	-
Immune checkpoint inhibitors-associated thrombosis in patients with head and neck cancer: a study of the Spanish Society of Medical Oncology (SEOM) thrombosis and cancer group [67]	Multicenter retrospective chart review	143	63	Male: 125 Female: 18	-	Head and neck cancer (100%)	2.8%	2.8%	Presence of liver metastasis
Association between immune checkpoints inhibitors with cardiovascular events and atherosclerotic plaque [68]	Single institution 2 study design: matched cohort and case cross-over	2842	64	Male: 1631 Female: 1211	-	NSCLC (28.8%) Melanoma (27.9%) Head and Neck (12.1%)	-	5%/year	ICIs Male sex Hypertension Diabetes

## Data Availability

No new data were created or analyzed in this study. Data sharing does not apply to this article.
